# Asymmetric Mating Interference between Two Related Mosquito Species: *Aedes* (*Stegomyia*) *albopictus* and *Aedes* (*Stegomyia*) *cretinus*

**DOI:** 10.1371/journal.pone.0127762

**Published:** 2015-05-22

**Authors:** Athanassios Giatropoulos, Dimitrios P. Papachristos, George Koliopoulos, Antonios Michaelakis, Nickolaos Emmanouel

**Affiliations:** 1 Laboratory of Biological Control of Pesticides, Department of Pesticides Control & Phytopharmacy, Benaki Phytopathological Institute, Kifissia, Athens, Greece; 2 Laboratory of Agricultural Zoology and Entomology, Agricultural University of Athens, Athens, Greece; 3 Laboratory of Agricultural Entomology, Department of Entomology and Agricultural Zoology, Benaki Phytopathological Institute, Kifissia, Athens, Greece; University of Crete, GREECE

## Abstract

*Aedes* (*Stegomyia*) *albopictus* (Skuse) and *Aedes* (*Stegomyia*) *cretinus* Edwards are closely related mosquito species with common morphological features and bio-ecological similarities. Recent mosquito surveillance in Athens, Greece, showed that they are sympatric mosquito species, with *Ae*. *Albopictus* developing quite higher population densities than *Ae*. *Cretinus*. The potential of mating interference between these species was investigated by reciprocal and homologous mating experiments in cages under laboratory conditions. In non-choice interspecific crosses (groups of males and females) females of both species produced sterile eggs. Insemination rate was 58% for *Ae*. *Cretinus* females and only 1% for *Ae*. *Albopictus* females. *Aedes albopictus* males were sexually aggressive and inseminated *Ae*. *Cretinus* females (31%) in choice experiments, where males of one species had access to mate with females of both species. Whereas, interspecific mating of *Ae*. *Albopictus* females with *Ae*. *Cretinus* males in the co-occurrence of *Ae*. *Cretinus* females was weaker (4%). *Aedes cretinus* females from non-choice crossing with *Ae*. *Albopictus* or *Ae*. *Cretinus* males were paired individually with conspecific males. The percentage of fertile *Ae*. *Cretinus* females was 17.5% when had encaged before with *Ae*. *Albopictus* males, compared to 100% when *Ae*. *Cretinus* females were encaged with conspecific males only. Probable ecological consequences of asymmetric mating between these ecologically homologous species in nature are discussed.

## Introduction

Competitive displacement principle describes the phenomenon of displacement or extinction of a species from another ecologically homologous species that shares the same ecological niche [1]. This type of displacement affects the range of species distribution and is the most severe outcome of interspecific competition in insects and arachnids, where both exploitation and interference mechanisms are implicated [2]. It has primarily been occurred between closely related species in cases where exotic species displayed native species or previously established exotic species, often due to human interventions, in the contest of biotic invasions or species introduction for biological control [2–4].

“Mating interference” or “satyrization” or “reproductive competition”, i.e. negative effects via interspecific mating depressing reproductive output, is one of the multiple mechanisms that may underlie the phenomenon of “competitive displacement” or “competitive reduction” in mosquitoes [4–7]. Asymmetric mating interference, whereby males of one species mate with a related species and produce inviable or less fit hybrid offspring or infertile eggs, has been proposed as a method for the biological control of pests and vectors [8] and as a mechanism that maintains parapatric distributions of related species in nature [5]. Mating interference may explain the parapatric distribution of certain tsetse flies in Africa [9], the dynamics of hybrid zones between tick species [10] and has been proposed as the cause for the parapatric distribution of the mosquitoes *Aedes aegypti* (L.) and *Ae*. *bahamensis* (Coquillett) on Grand Bahama Island [11].

*Aedes* (*Stegomyia*) *albopictus* (Skuse 1894), the so-called Asian tiger mosquito, is considered to be the most invasive mosquito species in the world possessing serious impact on biological diversity and human activities [12]. Among the invasive mosquito species detected in Europe, *Ae*. *albopictus* probably presents the major threat to public health [13]. It is a serious nuisance biting mosquito that is implicated in the transmission of a wide range of human pathogens, such as dengue and chikungunya virus and filarial nematods of the genus *Dirofilaria* [14]. Due to its quick and aggressive spread from its native range in East Asia and islands of the Western Pacific and Indian Ocean, *Ae*. *albopictus* has colonized every continent except Antarctica in the past 30–40 years [15]. Cross mating advantage of *Ae*. *albopictus* in reproductive competition with other native container breeding *Aedes* species has been considered among multiple hypotheses concerning the ecological processes operating during its invasions in several locations [6,7,16,17].

After its first detection in 2003–2004 in Northwestern Greece [18], *Ae*. *albopictus* has been subsequently found in several areas of the country [19]. A recent field work using ovitraps in the greater urban area of Athens showed that *Ae*. *albopictus* was widespread in the studied area performing considerably high ovipositioning activity, and revealed the presence of the indigenous mosquito *Aedes* (*Stegomyia*) *cretinus* (Edwards 1921) in negligible and restricted, however, population densities [20]. *Aedes albopictus* predominated in the well urbanized areas while *Ae*. *cretinus* was active only in the less crowded and more vegetated area where both species, however, co-occurred and maybe were given the opportunity to meet and interbreed [20]. *Aedes cretinus* shares many morphological and behavioral traits with *Ae*. *albopictus* [21], and both species have been placed into the subgroup *albopictus* (group *scutellaris*) of the subgenus *Stegomyia* [22]. The classification of these two *Aedes* species was revised by Reinert et al [23], though not generally accepted [24,25], so the subgenus of these two species under this new classification is currently uncertain. *Aedes cretinus* has a limited distribution across the world (Greece, Cyprus, Georgia and Turkey) and little appears to be known about its biology, while its capacity to transmit diseases has not been investigated [26]. It has been described as an aggressive day time biting tree-hole mosquito from wooded areas in Greece [27] and primarily wooded areas, open fields and road edges in Turkey [28,29].

Once a female mosquito has undergone a consummated union, usually becomes refractory to subsequent mating, at least until after completing a gonotrofic cycle. At a certain time after mating, the initial refractoriness of the female is reinforced by a pheromone, named matronae, which is contained in male accessory gland (MAG) secretions, transferred to her in semen fluid during copulation [26,30,31]. Induced female monogamy by MAG secretions that act either as a short-term mating plug physical barrier [32,33] or as a long-term chemical barrier to further insemination, has been shown to occur in *Aedes* mosquitoes [31,34–37] and particularly in *Ae*. *albopictus* [34,38,39]. Thus, mating interference between different mosquito species could sterilize a female that cannot produce viable offspring [40,41]. The tendency for *Ae*. *albopictus* to mate with other *Stegomyia* species, such as *Aedes polynesiensis* and *Ae*. *aegypti*, is well established [42–45]. Moreover, refractoriness in females to conspecific insemination in mosquito species after mating with *Ae*.*albopictus* males has been evidenced, either by cross mating experiments [46] or by interspecific implant of male accessory gland secretions [34,47].

The objective of this study was to determine if mating interference takes place between *Ae*. *albopictus* and *Ae*. *cretinus*. The potential of mating interference between these *Stegomyia* species was investigated by reciprocal and homologous mating experiments in cages under laboratory conditions. Since the results indicated asymmetric cross mating in favor of *Ae*. *albopictus* males, further experimentation was conducted in the lab in order to evaluate whether cross mating proficiency of *Ae*. *albopictus* males affects fertility of *Ae*. *cretinus*.

## Materials and Methods

### Mosquito rearing

Laboratory strains of *Ae*. *albopictus* and *Ae*. *cretinus* were established from eggs collected in June 2011, using ovitraps from areas of Rizoupoli (38°01’33”N, 23°44’28”E) and Chalandri (38°01’29”N, 23°48’18”E), respectively, located in the greater urban Athens area, Greece. Species discrimination was based on two easily distinguished differences on the scaling pattern of the scutum of female mosquitoes, according to identification key by Darsie and Samanidou-Voyadjoglou [21]. On *Ae*. *cretinus*’ scutum, submedian narrow lines of pale scales exist extending from just posterior to scutal angle to scutellum, and there are lateral lines of pale scales from anterior promotory to wing root. Instead, *Ae*. *albopictus*’ scutum has neither submedian nor lateral lines of pale scales. Colonies of both species were maintained in the laboratory at 26±2°C, 80% relative humidity, and photoperiod of 16:8-h (light:dark), in separate rooms of Benaki Phytopathological Institute, Kifissia, Greece. Adult mosquitoes were kept in wooden framed cage (33×33×33 cm) with a 32×32 mesh, with easy access to 10% sucrose solution on a cotton wick. Females were blood fed from senior author’s (AG) forearm. Larvae were reared in tap water-filled cylindrical enamel pans with diameter of 35 and 10 cm deep covered by fine muslin. Approximately 400 larvae were fed *ad libitum* with powdered fish food (JBL Novo Tom 10% Artemia) in each pan until the adults emerged. Adult mosquitoes were collected using mouth aspirator and transferred to the rearing cage. Plastic beakers with 100 ml water and strips of moistened filter paper were provided in the cage for oviposition. The eggs were kept wet for a few days and then placed in the pans for hatching.

### Cross mating studies

Cross matings were conducted in a laboratory room (26±2°C,80% relative humidity, and photoperiod of 16:8-h), using virgin males and females of each species, 2–3 days old, originated from the laboratory colonies. Pupae were kept individually in transparent plastic vials to ensure virginity in newly emerged adults. Adults of each sex and species were placed in wooden framed cage (33×33×33 cm) with a 32×32 mesh, with easy access to 10% sucrose solution on a cotton wick. To prevent accidental mating of encaged females through the screen by escaped males flying loose in the rearing room, cages were provided with a second layer of screen (muslin) separated by a space of 5 mm. Two trial series of non-choice and choice crosses between *Ae*. *albopictus* and *Ae*. *cretinus* adults were implemented.

In non-choice homologous and reciprocal crosses 20 males of one species were caged with 20 females either of the same or the other species. Moreover, 20 females alone were held in cages as egg laying control treatment. Five days after entering mosquitoes in the cages a human blood meal (AG) was employed until all females got blood fed. Plastic beakers with 100 ml water and strips of moistened filter paper were provided in the cage for oviposition. Ten days later the eggs laid were counted and maintained for five more days in moistened conditions to ensure embryogenesis. The eggs were hatched in dechlorinated water with powdered fish food applying two subsequent submerges and the emerged larvae were counted. All females were dissected and their spermathecae were examined for the presence of sperm to determine insemination rate. Spermathecae were placed in a drop of saline on a glass slide, covered with a cover slip, and gentle pressure was applied. The slides were then examined for the presence of sperm using phase-contrast illumination at 100x magnification [48]. For egg laying and egg hatching rate four cages, each containing 20 males and 20 females, were used for each homologous and reciprocal cross and virgin female treatment (20 females only). For insemination rate determination, the aforementioned four cages plus two more, thus six in total, each containing 20 males and 20 females were used as replicates.

In choice crosses males were provided with a choice of mate species. Twenty *Ae*. *albopictus* males were placed in a cage with 20 *Ae*. *albopictus* and 20 *Ae*. *cretinus* females. Similarly, 20 *Ae*. *cretinus* males were caged with 20 *Ae*. *albopictus* and 20 *Ae*. *cretinus* females. Adults were held in the cage with access to 10% sucrose for 15 days prior to dissection and determination of insemination rate. Species identification in females after choice cross was easily performed under stereoscope based on the two differences in the scaling pattern of the scutum [21]. When the contact of caged specimens caused loss of scales, we examined the distinguishing character in fore- and mid- tarsi; the claws are toothed in *Ae*. *cretinus*, whereas they are simple in *Ae*. *albopictus* [22]. Insemination rate was determined by phase-contrast microscopy, alike non-choice crosses. Each treatment was replicated six times.

### Reproductive interference of cross mating

To investigate reproductive interference of cross mating, 20 virgin males of *Ae*. *albopictus* were placed with 20 virgin females of *Ae*. *cretinus* with 10% sucrose in three cages under the same laboratory conditions that were described in cross mating studies. Mosquitoes were left to copulate in the cages for 15 days, and then *Ae*. *cretinus* females were transferred individually in 1.3 cm^3^ (10x10x13 cm) plastic cages, covered in the top with fine muslin, along with one virgin *Ae*. *cretinus* male, 2–3 days old, and sucrose 10%.

Five days later every pair was provided with a human blood meal (AG) and plastic beakers with 100 ml water and strips of moistened filter paper for oviposition. *Aedes cretinus* males and females were left to mate and oviposit for four weeks. Then the eggs were counted, hatched in dechlorinated water with powdered fish food following two subsequent submergences, and the emerged larvae were counted. As a control, virgin males and females of *Ae*. *cretinus* were treated following the same protocol under the same laboratory conditions, using new males for the subsequent pairs.

### Statistical analysis

In non-choice tests, effects of cross mating treatments, including virgin female controls, on number of eggs laid per cage were determined by one-way analysis of variance followed by a Student-Newman-Keuls post hoc test for comparison of mean number of eggs per cage between treatments (a = 0.05) [49]. Data concerning the effects of cross mating treatments on insemination rate were analyzed using Generalized Linear Model with Poisson distribution with loglink (a = 0.05) [50].

In choice tests, comparison of insemination rate between *Ae*. *albopictus* and *Ae*. *cretinus* females in each mating cross was performed using non parametric McNemar test for related samples (a = 0.05) [50].

In reproductive interference study, *x* ^2^ test (a = 0.05) was performed to compare the percentage of *Ae*. *cretinus* fertile females that had previously mated with *Ae albopictus* males and then paired with conspecific males (treatment) with those mated with conspecific males only (control). Non-parametric Mann—Whitney U tests were carried out for pair-wise comparisons of *Ae*. *cretinus* eggs laid and percentage of hatched larvae between treatment and control (a = 0.05) [49].

All analyses were conducted using the statistical package SPSS 14.0 [51].

### Ethics statement

For establishment of laboratory mosquito strains no specific permits were required to collect mosquito eggs using ovitraps from areas in Athens, Greece, because they are public areas and are not privately owned or protected. Mosquito egg collections from the field did not involve endangered or protected species. Blood meals were provided to mosquitoes by the senior author’s forearm (AG) for experimental purposes with his full consent following medical treatment by applying appropriate anti-pruritic skin gel. An informal ethical group consisting of the Director of the Institute and two members of the Institutional Scientific Council was established and determined that human blood feeding of mosquitoes did not involve human as research subject, thus was not a subject to review.

## Results

**Fig 1:**
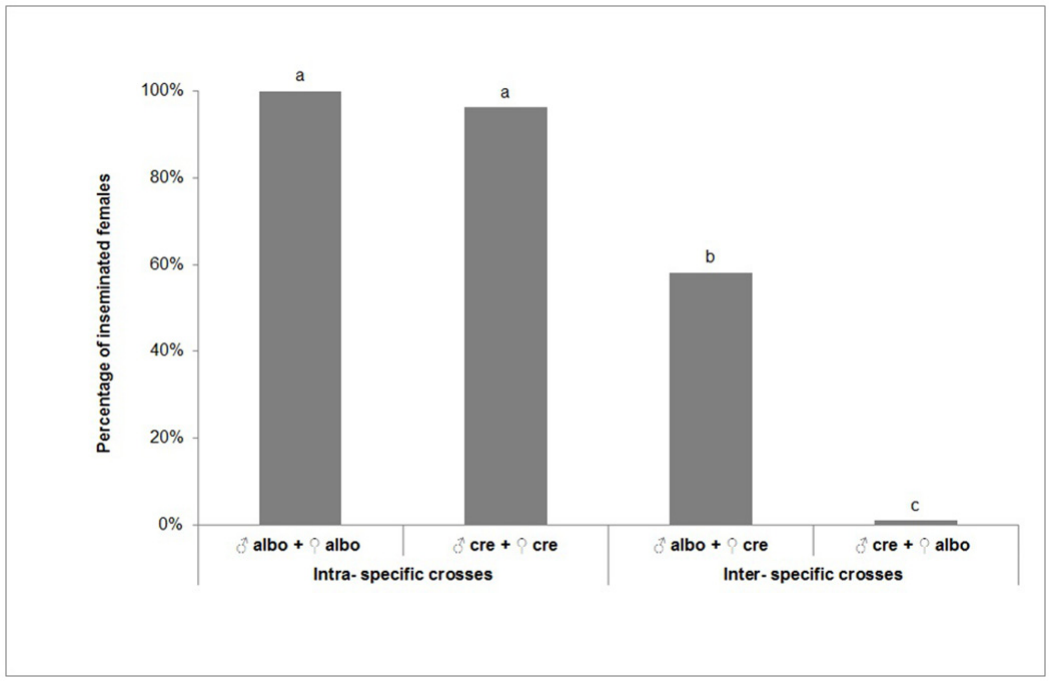
Percentage (%) of inseminated females after intra- and inter- specific crosses of 20 virgin *Ae*. *albopictus* and *Ae*. *cretinus* males and females. *Percentages in a column followed by different letter are significantly different (*P*< 0.05), 95% Wald Confidence Interval. ** “albo” = *Aedes albopictus*, “cre” = *Aedes cretinus*.

In non-choice cross mating studies the number of eggs laid was significantly affected by the cross mating treatment (F = 45.541; d.f. = 5, 18; *P*<0.0001) (Table 1). The average number of eggs laid were significantly different between homospecific and heterospecific crosses. Crossing of *Ae*. *cretinus* females with *Ae*. *albopictus* males resulted in significantly higher number of eggs compared with the reciprocal crossing. All eggs produced by heterologous crosses were sterile (Table 1). In non-choice tests insemination rate differed significantly between the treatments (Likelihood *x* ^2^ = 428.012; d.f. = 3; *P*<0.0001) and was very high (100 and 96%) in case of intraspecific crosses. Interestingly, in interspecific crosses a considerable percentage of *Ae*. *cretinus* females was inseminated (58%) compared with the very low insemination rate for *Ae*. *albopictus* (1%) (Fig 1).

**Table 1:** Mean number of eggs (± S.E.M) and mean percentage of hatched larvae (± S.E.M) per cage of intra- and inter- specific non choice crosses of 20 virgin *Ae*. *albopictus* and *Ae*. *cretinus* males and females.

Cross mating	Replicates (cages)	Mean number of eggs per cage (±S.E.M)*	Mean percentage (%) of hatched larvae per cage (±S.E.M)
*♂ Ae*. *albopictus + ♀ Ae*. *albopictus*	4	498±38**a**	94±2
*♂ Ae*. *cretinus + ♀ Ae*. *cretinus*	4	314±54**b**	87±4
*♂ Ae*. *albopictus + ♀ Ae*. *cretinus*	4	213±28**c**	0
*♂ Ae*. *cretinus + ♀ Ae*. *albopictus*	4	36±11**d**	0
*♀ Ae*. *albopictus*	4	11±8**d**	0
*♀ Ae*. *cretinus*	4	2±2**d**	0

*Means in a column followed by different letter are significantly different (SNK test, *P*<0.05).

**Fig 2:**
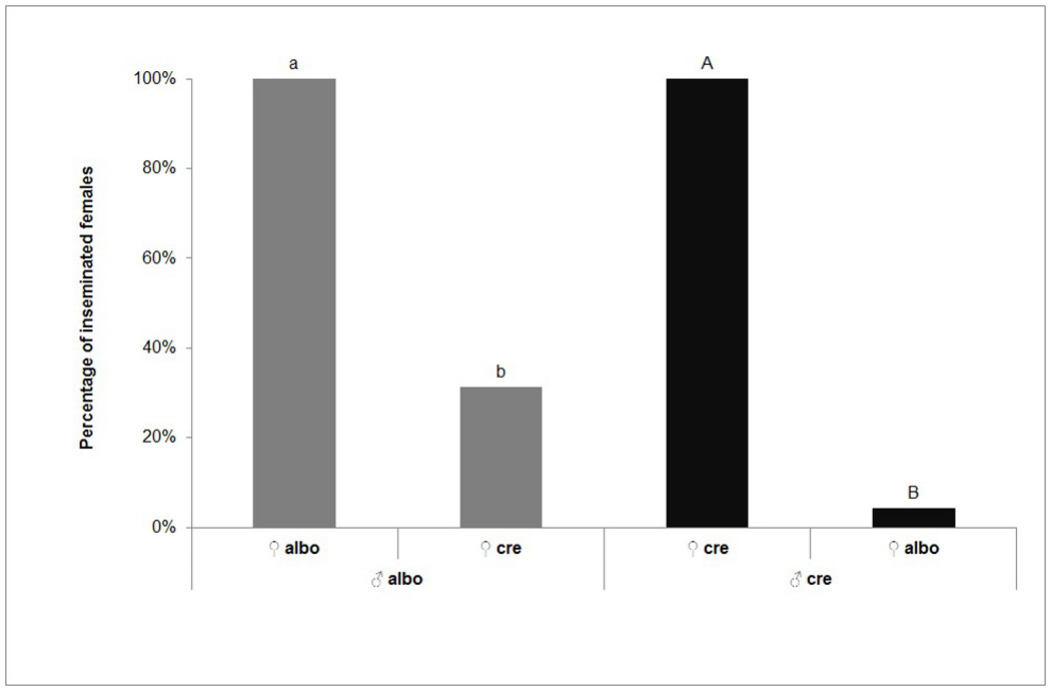
Percentage (%) of inseminated females after choice crosses of 20 virgin males of *Ae*. *albopictus* or *Ae*. *cretinus* with 20 *Ae*. *albopictus* and 20 *Ae*. *cretinus* virgin females. *Percentages in a column followed by different small or capital letter are significantly different (*P*< 0.05, McNemar test for related samples). ** “albo” = *Aedes albopictus*, “cre” = *Aedes cretinus*.

In choice tests for *Ae*.*albopictus* males, insemination rate was significantly higher for *Ae*. *albopictus* females (100%) compared with that for *Ae*. *cretinus* females (31%) (*x* ^*2*^ = 64.015; *P*< 0.0001). Accordingly, in case of encaged *Ae*. *cretinus* males with females of both species, percentage of inseminated *Ae*. *cretinus* females (100%) was significantly higher than that of *Ae*. *albopictus* females (4%) (*x* ^*2*^ = 89.011; *P*<0.0001) (Fig 2).

Table 2 shows that in reproductive interference study, *Ae*. *cretinus* females paired individually with conspecific males laid eggs, either with previous crossing with *Ae*. *albopictus* (67.6±3.7) or *Ae*. *cretinus* (77.3±1.5) males and mean numbers of eggs did not differ significantly (Z = -1.512; *P* = 0.131). However, the percentage of fertile *Ae*. *cretinus* females paired with *Ae*. *cretinus* males individually, after had been crossed with *Ae*. *albopictus* males (17.5%), was significantly lower (*x* ^2^ = 77.225; d.f. = 1; *P*<0.0001) compared with fertile *Ae*. *cretinus* females that were encaged with conspecifics only (100%). Mean number of eggs laid and percentage of hatched larvae by fertile *Ae*. *cretinus* females were not significantly affected by cross mating with *Ae*. *albopictus* males (Z = -1.240; *P* = 0.215 and Z = -1.590; *P* = 0.112, respectively)

**Table 2:** Oviposition and fertility of *Ae*. *cretinus* females previously crossed with *Ae*. *albopictus* (treatment) or *Ae*. *cretinus* (control) males in groups of 20 and then paired with *Ae*. *cretinus* males individually.

	*Aedes cretinus* females					
	Total		Fertile ♀			
	No. **♀**	Mean No. of eggs per **♀** (±SEM)**	No. **♀**	Percentage (%)*	Mean No. of eggs (±SEM)**	Mean % of hatched larvae (±SEM)**
**Treatment**	57	67.6±3.7a	10	17.5a	69.4±7.6a	69.5±13.6a
**control**	54	77.3±1.5a	54	100b	77.3±1.5a	90±1.7a

* Percentages with different letters are significantly different (*P*< 0.05, *x* ^2^ test).

** Means in a column followed by the same letter are not significantly different (*P*>0.05, Mann—Whitney U test).

## Discussion

In the current work a quite high insemination rate of *Ae*. *cretinus* females was recorded after heterospecific non-choice crosses with *Ae*. *albopictus* males. The ability of *Ae*. *albopictus* males to mate with *Ae*. *cretinus* females was confirmed in choice experiments. Interestingly, this cross mating behavior for the two species performed in an asymmetric pattern, since *Ae*. *cretinus* males mated in negligible rate with *Ae*. *albopictus* females throughout reciprocal crosses either in non-choice or choice treatments. Structural incompatibilities of genitalia, differing responses of males to females’ flight sound and both the ability to produce and recognize semiochemicals have been identified as essential parameters in *Aedes* interspecific mating [31,40,52,53,54] and therefore may account for asymmetry in cross mating between *Ae*. *albopictus* and *Ae*. *cretinus*. Cross mating proficiency of *Ae*. *albopictus* males with other *Stegomyia* mosquito species has been reported by several authors. Results of cage experiments by Nasci et al [43] and Bargielowski et al [45] suggested that *Ae*. *aegypti* females are more receptive to insemination by *Ae*. *albopictus* males than *Ae*. *albopictus* females are receptive to insemination by *Ae*. *aegypti* males. Nazni et al [44] reported successful bidirectional cross-mating between *Ae*. *albopictus* and *Ae*. *aegypti*, which was followed by oviposition of sterile eggs. Cross mating between *Ae*. *albopictus* males and *Ae*. *polynesiensis* females occurred readily under simulated natural conditions in a large cage, where *Ae*. *albopictus* males and females of both sexes were engaged, during the course of a long term competition experiment [42].

Interspecific non-choice crosses between *Ae*. *albopictus* and *Ae*. *cretinus* in both directions produced eggs, but without progeny-hybrid outcome. Infertile cross mating between *Aedes* members has been observed by Leahy and Craig [40] who worked with multiple laboratory colonies and found that no hybrid offspring was produced in interspecific crosses of *Ae*. *albopictus* and *Ae*. *aegypti*. Sterile eggs from cross mating were significantly higher in numbers when *Ae*. *albopictus* males crossed with *Ae*. *cretinus* females. High ovipositioning by *Ae*. *cretinus* in cross mating correlates with the high insemination rate and may be attributed to stimulant substance provided by the male accessory gland. According to Leahy and Craig [55] implants of heterologous male accessory glands to *Ae*. *albopictus* and *Ae*. *aegypti* females resulted in ovipositioning of sterile eggs indicating that upon mating a substance stimulus to egg deposition is provided by the male accessory glands.

*Aedes* females have been observed to copulate several times in their life time [56], and though they are considered as primarily monandrous, examples of multiple insemination (polyandry) exist both in the laboratory and in the field [57–59]. Thus, no adverse effect of the presence of heterospecific males on reproductive success was found in choice laboratory tests for either *Ae*. *albopictus* or *Ae*. *aegypti* [60,61]. The critical question, therefore, is not only whether cross mating occurs, but rather, whether heterospecific mating interferes with conspecific mating and oviposition [61]. The experimental evidence of the current reproductive interference study suggests that the cross inseminated *Ae*. *cretinus* females cannot be re-inseminated by conspecific males and therefore *Ae*. *albopictus* males appeared highly effective in sterilizing *Ae*.*cretinus* females. Such reproductive interference has been observed under laboratory conditions, where males of *Ae*. *albopictus* mated readily with the females of *Ae*. *polynesiensis* and these females did not produce viable eggs after engagement with conspecific males [46]. Also, Tripet et al [47] injected heterologous MAG in *Ae*. *aegypti* and *Ae*. *albopictus* virgin females and reported sterilization for the former species, but no effect on the ability of *Ae*. *albopictus* to mate with their own species. In a publication by Craig [34] heterologous implants of MAG from *Ae*. *atropalpus*, *Ae*. *triseriatus*, *Culex pipiens*, *Ae*. *scutellaris* and *Drosophila melanogaster* to *Ae*. *aegypti* females and from *Ae*. *aegypti* and *Ae*. *atropalpus* to *Ae*. *triseriatus* females significantly affected insemination rate with conspecific males.

Theoretically, if asymmetric mating interference between *Ae*. *albopictus* and *Ae*. *cretinus* that was evidenced under laboratory conditions, apply in nature it may affect population dynamics of these ecologically homologous species, causing even displacement of *Ae*. *cretinus*. Ribeiro [8] suggests that the rate and degree of displacement due to satyrization depends upon the relative density, reproductive and dispersal rates, and on the degree of asymmetry in mating aggressiveness between the two species. The ecological consequences of “reproductive interference”, a term for several types of sexual interactions between animal species including heterospecific mating, can be dramatic leading to displacement of one species (sexual exclusion), spatial, temporal, or habitat segregation, changes in life history parameters, and reproductive character displacement [62]. Heterospecific matings and hybridization are often considered to be the types of reproductive interference with the highest fitness losses, as they involve gamete wastage and can lead to sterile offspring or inviable eggs [62]. Our experiments revealed a strong asymmetry in cross mating favoring satyrization of *Ae*. *cretinus* by *Ae*. *albopictus* and causing detrimental genetic material waste for *Ae*. *cretinus* through the production of sterile eggs. Considerably high population densities of *Ae*. *albopictus* against low ones of *Ae*. *cretinus*, that have been recorded in our field study in Athens [20], provide probably *Ae*. *albopictus* with a competitive advantage in the vegetated areas where these species co-exist.

A recent and well-studied example of competitive displacement or reduction between mosquito species was the rapid reduction in range and abundance of *Ae*. *aegypti* following the invasion and spread of *Ae*. *albopictus* throughout most of the southeastern United States in the 1980s [16,17,63–65]. Laboratory and field data indicate that asymmetric mating interference triggering sterility to *Ae*. *aegypti* could constitute a potential explanation for this displacement [41,43–45,47,66]. Interestingly, exposures in cages demonstrated that female *Ae*. *aegypti* from populations in Florida sympatric with *Ae*. *albopictus* for the past 20 years were significantly less likely than nearby allopatric populations to mate with heterospecific males [45]. Those results indicate rapid sexual selection leading to reproductive character displacement and the potential for satyr-resistant *Ae*. *aegypti* to recover from competitive displacements [45]. *Aedes albopictus* and *Ae*. *cretinus* have been detected as sympatric species in vegetated areas of Athens [20], however *Ae*. *cretinus* satyr-resistance was not observed in the current study. This might happened because mosquito populations that were used to establish laboratory colonies were allopatric and therefore reproductive isolation had not evolved.

Overall, the current laboratory cross mating studies provided evidence for asymmetric mating interference between *Ae*. *albopictus* and *Ae*. *cretinus* in favor of *Ae*. *albopictus*. It is essential to look for possible explanations for this cross mating asymmetry, identifying for example structural compatibilities of genitalia and the role of cues that may be involved in sexual interaction between these species. Further field work is deemed necessary to study if mating interference may account for the limited presence of *Ae*. *cretinus* and the extended one of *Ae*. *albopictus* in the urban environment of Athens, Greece where these related species co-occur.
