# Correction: Asymmetric Mating Interference between Two Related Mosquito Species: *Aedes (Stegomyia) albopictus* and *Aedes (Stegomyia) cretinus*

**DOI:** 10.1371/journal.pone.0132862

**Published:** 2015-07-06

**Authors:** The PLOS ONE Staff

In the Abstract, the names of the species are incorrectly listed. All instances of *Ae*. *Albopictus* should be *Ae*. *albopictus* and all instances of *Ae*. *Cretinus* should be *Ae*. *cretinus*. The publisher apologizes for the error.
